# Macrophage Plasticity and Atherosclerosis Therapy

**DOI:** 10.3389/fmolb.2021.679797

**Published:** 2021-05-07

**Authors:** Ping Lin, Hong-Hai Ji, Yan-Jie Li, Shou-Dong Guo

**Affiliations:** Institute of Lipid Metabolism and Atherosclerosis, Innovative Drug Research Centre, School of Pharmacy, Weifang Medical University, Weifang, China

**Keywords:** macrophage polarization, plaque microenvironment, inflammation, lipid metabolism, ER stress, apoptosis

## Abstract

Atherosclerosis is a chronic disease starting with the entry of monocytes into the subendothelium and the subsequent differentiation into macrophages. Macrophages are the major immune cells in atherosclerotic plaques and are involved in the dynamic progression of atherosclerotic plaques. The biological properties of atherosclerotic plaque macrophages determine lesion size, composition, and stability. The heterogenicity and plasticity of atherosclerotic macrophages have been a hotspot in recent years. Studies demonstrated that lipids, cytokines, chemokines, and other molecules in the atherosclerotic plaque microenvironment regulate macrophage phenotype, contributing to the switch of macrophages toward a pro- or anti-atherosclerosis state. Of note, M1/M2 classification is oversimplified and only represent two extreme states of macrophages. Moreover, M2 macrophages in atherosclerosis are not always protective. Understanding the phenotypic diversity and functions of macrophages can disclose their roles in atherosclerotic plaques. Given that lipid-lowering therapy cannot completely retard the progression of atherosclerosis, macrophages with high heterogeneity and plasticity raise the hope for atherosclerosis regression. This review will focus on the macrophage phenotypic diversity, its role in the progression of the dynamic atherosclerotic plaque, and finally discuss the possibility of treating atherosclerosis by targeting macrophage microenvironment.

## Introduction

Despite advances in plasma cholesterol-lowering, cardiovascular disease (CVD) remains the major cause of death worldwide (Zhao et al., 2019; Timmis et al., 2020). Atherosclerosis is a progressive disease and the key underlying cause of CVD events (Libby, 2002; Guo et al., 2020). Atherosclerosis mainly affect large and medium-sized arteries and is characterized by the formation of atherosclerotic plaques which are composed of lipids, necrotic cores, calcified regions, inflamed smooth muscle cells (SMCs), endothelial cells, immune cells and foam cells (Tabas and Bornfeldt, 2016; Shapouri-Moghaddam et al., 2018).

It has been acknowledged that atherosclerosis is a chronic inflammatory disease (Ross, 1999). During inflammation, circulating monocytes migrate into the endothelium from the blood flow and then differentiate into macrophages when exposed to the local microenvironment that is enriched in growth factors and pro-inflammatory cytokines (Narasimhan et al., 2019; Kim et al., 2020). The earliest atherosclerotic lesion, fatty streak, is associated with an increased number of intimal macrophages and the appearance of macrophages filled with lipid droplets (foam cells) (Stary et al., 1994). In humans, the intermediate lesions are characterized by pools of extracellular lipid and the accumulation of foam cells that derived from macrophage and SMCs in the artery wall (Stary et al., 1994). Cycles of accumulation of foam cells, local necrosis, and formation of fibrous cap lead to the formation of stable or unstable plaques (Stary et al., 1995). Stable plaques are characterized by a relatively small lipid core enclosed by a thick fibrous cap, whereas unstable plaques are mostly characterized by a large lipid core covered by a thin fibrous cap that prone to rupture (Stary et al., 1995; Fuster et al., 1999).

Macrophages are the central cells in atherosclerotic plaques and account for the majority immune cells (Moroni et al., 2019). To better understand the role of these cells, macrophage classification was first provided in the year of 1962 (Mackaness, 1962). In the following years, M1 macrophages have been extensively studied until the presence of M2 macrophages. Previously, it is accepted that M1 macrophages initiate and sustain inflammation, whereas M2 macrophages counteract the inflammation (Chinetti-Gbaguidi et al., 2014; Nagenborg et al., 2017; Jinnouchi et al., 2020). However, the concept of macrophage heterogeneity within the atherosclerotic plaques has gradually emerged during the past decades. It is worth noted that single-cell technologies have further expanded our knowledge about cell phenotypic diversity (Spitzer and Nolan, 2016; Jinnouchi et al., 2020). These novel data have challenged the established classification. Now, M1/M2 classification is considered as oversimplified and only represent two extreme states of macrophage. More importantly, macrophages can switch from one phenotype to another upon microenvironmental stimuli, and M2 phenotype is not always protective (Guo L. et al., 2018; Pourcet and Staels, 2018). Although macrophage phenotypic diversity within atherosclerotic plaques remains controversial, recent new progress in this field provide potential therapeutic strategies for treatment of atherosclerosis by targeting plaque macrophage microenvironment.

## Macrophage Phenotypic Diversity

In atherosclerotic plaques, macrophages are derived from infiltrating monocytes and proliferating vascular resident macrophages (Robbins et al., 2013; Zernecke et al., 2020). In mouse, short-lived monocyte-derived macrophages are characterized by high expression of Ly6C, CC chemokine receptor (CCR) 2, and cluster of differentiation (CD) 11b, whereas long-lived tissue-resident macrophages can be of embryonic origin or derive from circulating monocyte intermediates and are characterized by high expression of F4/80, CD64, MerTK, and CD14 (Gentek et al., 2014; Trzebanski and Jung, 2020). However, monocyte contribution might gradually increase with age due to the decreased capacity of self-renewal potential of the resident macrophages. It is worth noting that macrophages of embryonic origin and monocytes have distinct preferences of differentiation toward MHCII^+^ and MHCII^–^ subsets, suggesting their potential distinct functions. Furthermore, the alterations of macrophage origin may change the dominant macrophage subpopulation along with a functional alteration (Gentek et al., 2014; Honold and Nahrendorf, 2018). Accumulating evidence suggest that microenvironment as well as the origin of macrophages may finally determine the function of the cells.

The plasticity of monocyte development and monocyte fates have been reviewed recently (Italiani and Boraschi, 2014; Hilgendorf et al., 2015; Trzebanski and Jung, 2020). In general, Ly6C^*high*^ monocytes in mice are similar to the human classical CD14^++^/CD16^–^ subtype which represent the majority of circulating monocytes (∼90%), whereas Ly6C^*low*^ monocytes are similar to the human non-classical CD14^+^/CD16^++^ subtype monocyte. Although monocytes are rare in healthy arteries, studies have demonstrated that atherosclerosis is associated with the level of CD16^+^ monocyte (Schlitt et al., 2004; Rogacev et al., 2012; Nagenborg et al., 2017). Furthermore, CD14^++^/CD16^–^ monocytes are associated with inflammation and a thinner fibrous cap (Berg et al., 2012). These data suggest that monocytes are a risk factor of atherosclerosis. Dislipidemia and inflammation can trigger the recruitment and retention of monocytes in the damaged arterial wall (Nagenborg et al., 2017; Rahman M. S. et al., 2017). Of note, monocyte progenitor cells with defective cholesterol efflux capacity are associated with an elevated monocytosis and atherosclerosis (Yvan-Charvet et al., 2010). Monocyte-endothelial cell adhesion is mediated by complicated signaling pathways, such as the interaction between P-selectin expressed on damaged endothelial cells and the special glycosylated P-selectin glycoprotein ligand-1 that expressed on monocytes (Elstad et al., 1995; McEver et al., 1995). The interaction of monocyte integrins (such as very-late antigen 4) with vascular cell adhesion molecule 1 (VCAM-1) or intercellular cell adhesion molecule-1 (ICAM-1) that expressed on activated endothelial cells forms a strong adhesion between monocytes and endothelial cells (Xu et al., 2019). In the following, monocytes penetrate through endothelial cells into the subendothelial space and display a reduced migration ability.

Within the intima, monocytes differentiate into macrophages by multiple prodifferentiation factors such as macrophage colony-stimulating factor (M-CSF and CSF-1) and other differentiation factors such as IL-34 (Franzoni et al., 2017; Boulakirba et al., 2018; Xu et al., 2019). Upon stimulus of inflammation and lipoprotein retention, Ly6C^*high*^ monocytes are rapidly recruited to sites of inflammation and differentiate into monocyte-derived macrophages in mice. These macrophages scavenge lipoprotein particles and turn into foam cells, which form early atherosclerotic plaques and further promote lipoprotein retention by inducing a cascade of inflammatory responses. Furthermore, hematopoietic stem and progenitor cells migrate to the spleen and enhance the generation of splenic monocytes, such as Ly6C^*high*^ subtype, which display inflammatory properties and infiltrate the atherosclerotic plaque, contributing to the deterioration of atherosclerosis (Robbins et al., 2012; Dutta et al., 2012; Nagenborg et al., 2017). In general, Ly6C^*high*^ monocytes derived from bone marrow or extramedullary haemotapoiesis are the major precursor of plaque macrophages in early plaques, and their recruitment is dependent on CCR2, CCR5, and CX3CR1 (Combadiére et al., 2008; Nagenborg et al., 2017). It needs to point out that some monocytes may differentiate into monocyte-derived dendritic cells or just remain monocytes (Zernecke et al., 2020). It is suggested that the depletion of monocytes may inhibit atherosclerosis in early stage but not in advanced stage of atherosclerotic plaques (Stoneman et al., 2007). Interestingly, Rahman K. et al. (2017) found that the mostly reparative macrophages in regressing lesions may originate from the newly recruited Ly6C^*high*^ monocytes. Additionally, Ly6C^*low*^ monocytes are suggested to be associated with inflammation resolution (Moore and Tabas, 2011). Classical monocytes with proinflammatory features are the majority of total monocytes, while non-classical monocytes display more M2-like properties and may counterbalance the effects of classical monocytes. Furthermore, the intermediate CD14^++^/CD16^+^ subtype monocyte account for ∼5% of the total monocytes and have a stronger capacity to adhere to endothelium than the classical and non-classical monocytes (Foster et al., 2013). An interesting hypothesis is that different monocyte subsets may differentiate into distinct macrophages, thereby contributing to the formation of corresponding plaques with different vulnerabilities (Xu et al., 2019). Although these inflammatory Ly6C^*high*^ monocytes predominantly contribute to the early atherogenesis, replenishment of macrophages in established plaques is mainly depended on local resident macrophage proliferation rather than monocyte influx (Robbins et al., 2013; Rahman K. et al., 2017).

As reviewed by Sieweke and Allen, both yolk sac progenitor cells and monocyte-derived macrophages contribute to resident macrophages. Furthermore, resident macrophages are often F4/80^*high*^, whereas recruited monocyte-derived macrophages are generally F4/80^*low*^ (Sieweke and Allen, 2013). Tissue-resident macrophages are present in all major organs including arteries. These cells can proliferate at low levels in steady-state conditions and display high proliferation rate under inflammatory challenge. These tissue-resident macrophages show distinct functions depending on different tissue microenvironment (Italiani and Boraschi, 2014; Nahrendorf, 2019). Of note, lesional macrophages may proliferate and locally augment their numbers in plaques, independently of input from adult hematopoietic stem cells (Robbins et al., 2013; Sieweke and Allen, 2013). In mice, arterial macrophages arise embryonically from CX3CR1^+^ precursors and postnatally from circulating monocytes. In adulthood, resident arterial macrophages are maintained by CX3CR1-CX3CL1 interactions and local proliferation rather than monocyte recruitment (Ensan et al., 2016). The study of Psaltis et al. (2014) suggested that vessel wall serves as a source of tissue-resident macrophages. Sca-1^+^CD45^+^ adventitial macrophage progenitor cells are not replenished via the circulation monocytes from bone marrow or spleen. These cells are upregulated in hyperlipidemic atherosclerosis mouse models and contribute to macrophage progeny particularly in the adventitia, and to a lesser extent the atheroma, of atherosclerotic carotid arteries (Psaltis et al., 2014). Many cytokines, such as interleukin (IL)-4, IL-13, and M-CSF, and other cues such as damage-associated molecular patterns and pathogen-associated molecular patterns, mediate the resident macrophage self-renewal and determine the spectrum of tissue macrophages (Sieweke and Allen, 2013; Gentek et al., 2014; Rahman K. et al., 2017). Furthermore, dyslipidemia might lead to increased proliferation of tissue-resident macrophages (Rahman M. S. et al., 2017). A study demonstrated that inhibition of plaque neovascularization can reduce macrophage accumulation and progression of advanced atherosclerosis (Moulton et al., 2003). Differences in resident macrophage phenotypes can influence their capacity to proliferate locally, contributing the potential alteration of the abundant macrophage phenotype (Jenkins et al., 2011; Jenkins et al., 2013). As reviewed by Nahrendorf, tissue-resident macrophages are supposed to display various roles: (1) they inhibit tissue inflammation; (2) they rapidly alert the immune system when encountering infection; (3) they regulate the matrix metabolism by interacting with fibroblasts and by producing proteases that degrade extracellular matrix (Hulsmans et al., 2018; Meschiari et al., 2018).

In general, monocytes/macrophages are essential for the inflammatory response to microbes (Italiani and Boraschi, 2014). T helper 1 (Th1) activated M1 macrophages are responsible for cellular immunity to infection (Mills et al., 2000; Jinnouchi et al., 2020). Activation of macrophages by Th2 cytokines leads to the polarization of M2 macrophages (Gordon, 2003; Mantovani et al., 2004). Macrophage polarization is a process that macrophages gradually mount a phenotype with specific functions in response to a microenvironmental stimulus (Sica and Mantovani, 2012). Theoretically, distinct macrophage subtypes can be identified by their characteristic expression of surface markers and chemokine receptors. However, some markers are shared by different macrophage subtypes and only a few markers are specific for a given phenotype (Chinetti-Gbaguidi et al., 2014). The potential overlap of these markers increases the complexity of macrophage phenotype classification. For this reason, macrophage phenotypes are generally defined based on their surface markers as well as their possible functions. Monocytes, monocyte-derived macrophages, and tissue-resident macrophages comprise heterogeneous cell populations that adapt their functional phenotype in response to local microenvironmental stimuli (Geissmann et al., 2003; Gordon and Taylor, 2005).

Within the atherosclerotic plaques, monocyte-derived macrophages can polarize into different subtypes with distinct phenotypes and functions. Upon activation with interferon (IFN)-γ, tumor necrosis factor (TNF), and toll-like receptor (TLR) ligands, such as lipopolysaccharide (LPS), monocytes/macrophages exhibit a typical pro-inflammatory M1 phenotype (Shioi and Ikari, 2018; Jinnouchi et al., 2020). IFN-γ-Jagged1 axis may play a key role in monocytes differentiation toward an M1 phenotype (Kibbie et al., 2016). Of note, hemolytic environment and specifically the hemoglobin-activated platelets differentiate monocytes into pro-inflammatory M1-like macrophages (CD80^*high*^) (Singhal et al., 2018). Immune complexes from patients with systemic autoimmune diseases favor the polarization of monocyte-derived macrophages into a proinflammatory M1-like macrophages (Burbano et al., 2019). Some endogenous chemical compounds may play roles in monocytes differentiation. For instance, calcium oxalate can differentiate human monocytes into inflammatory M1 macrophages (Dominguez-Gutierrez et al., 2018), and less stable intravenous iron preparations may also affect monocyte differentiation toward macrophages (Fell et al., 2016). microRNAs also participate monocytes differentiation through regulating transcription factors in response to the microenvironment signals (Li et al., 2018). For instance, miR-148a-3p may promote monocyte differentiation and M1 macrophage activation through Notch signaling (Huang et al., 2017). The activated M1 macrophages secrete reactive oxygen species (ROS) and nitric oxide (NO) due to the activation of NADPH oxidase system, pro-inflammatory cytokines, such as IL-1β, IL-6, IL-12, IL-23 and TNFα, Th1 recruitment-associated chemokines, such as chemokine (C-X-C motif) ligand (CXCL)-9, CXCL-10, and CXCL-11, and low levels of anti-inflammatory cytokine IL-10 (Gordon, 2003; Domschke and Gleissner, 2019). Therefore, activation of M1 macrophage induces tissue damage and impairs wound healing (Murray and Wynn, 2011). On the contrary, activation of M2 macrophages is responsible for suppressing inflammation, scavenging cell debris and apoptotic cells, contributing to tissue repair and fibrosis (Chinetti-Gbaguidi et al., 2011; Murray and Wynn, 2011; Zizzo et al., 2012). Th2-associated cytokines, such as IL-4, IL-13, induce monocytes/macrophages toward M2 macrophages, and these cells are characterized by the expression of CD163, mannose receptor 1, resistin like-β, and high levels of arginase-1 (Barrett, 2020). For instance, both monocyte-derived and tissue-resident macrophages can be induced to proliferate by IL-4 (Jenkins et al., 2011). Some enzymes play important roles in monocyte/macrophage polarization. Matrix metalloproteinase (MMP)-8 may induce monocytes toward an M2 phenotype by cleaving fibromodulin and thereby enhancing the levels of transforming growth factor-β (TGF-β) (Wen et al., 2015). Monoamine oxidase A play a key role in activation of monocyte/macrophages toward an M2 phenotype (Cathcart and Bhattacharjee, 2014). Additionally, virus also play important roles in monocyte differentiation. For instance, hepatitis C virus may induce monocyte differentiation into polarized M2 macrophages (Saha et al., 2016; Zhang et al., 2016). From a metabolic point of view, M2 polarization is dependent on oxidation of fatty acid that produced via hydrolysis of triacylglycerol substrates within macrophages (Huang et al., 2014). Upon activation, these cells secrete high levels of anti-inflammatory cytokines, such as IL-10 and TGF-β, and chemokines as well as low levels of inflammatory cytokine IL-12 (Mantovani et al., 2004). Zarif et al. (2016) established a phased strategy to differentiate human CD14^+^ monocytes into M1 and M2 macrophages by alterations in cytokine composition, dosing, and incubation times, *in vitro*. Moreover, the authors demonstrated that M2 macrophages induced *in vitro* can express high levels of functional mannose receptor (CD206), which is an endocytic receptor (Zarif et al., 2016).

**FIGURE 1:**
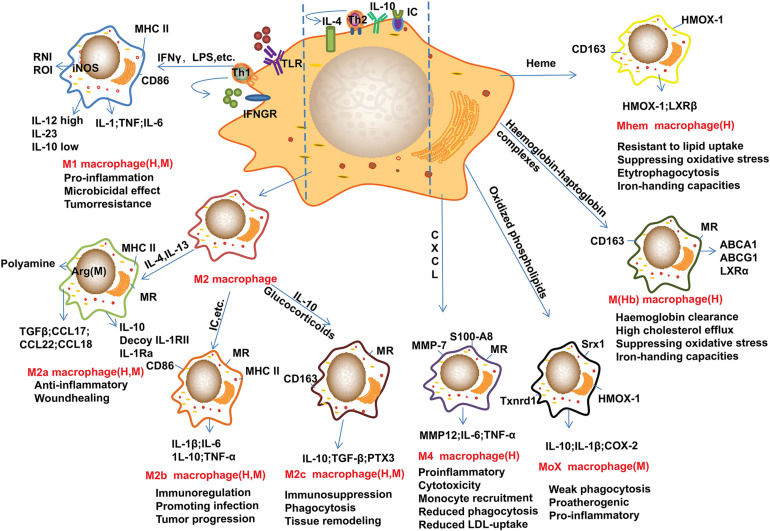
Main subtypes of macrophages found in atherosclerotic plaques. Distinct stimuli in atherosclerotic microenvironment drive distinct macrophage phenotype differentiation. This figure points out some examples of these macrophage markers or related genes, and the subtypes of macrophages are identified in human (H) and/or mouse (M) atherosclerotic plaques. M1 macrophages can be induced by IFN-γ and LPS, these cells mainly show pro-inflammatory effect. M2 macrophages can be induced by IL-4 and IL-13, and IL-10, these cells can be further divided into M2a, M2b, and M2c subtypes. Of note, these cells are found not always protective. Furthermore, monocyte/macrophage can be differentiated into Mox, M4, M(Hb), and Mhem subtypes by distinct factors or microenvironments. Arg-1, arginase-1; CD, cluster of differentiation; CXCL, Chemokine (C-X-C motif) ligand; HMOX-1, heme oxygenase-1; IC, immune complexes; iNOS, inducible nitric oxide synthase; IFN-γ, interferon-γ; IL, interleukin; LPS, lipopolysaccharide; LXR, liver X receptor; MHC, major histocompatibility complex; MMP, Metalloproteinase; MR, mannose receptor; Srx1, sulforedoxin-1; Th, T-helper; Txnrd1, thioredoxin reductase 1; TLR, Toll-like receptor; TGFβ, transforming growth factor β. These abbreviations are suitable for the following figures.

Recent advances suggested that M2 macrophages can be further divided into several subtypes as shown in Figure 1. For example, lipoxin-A (4) and annexin-A1 induce monocyte differentiation into M2a + M2c-like cells through modulating the phosphorylation of signal transducers and activators of transcription (STAT) 3 in a formyl peptide receptor-2 dependent manner (Li et al., 2011). Of note, M2d macrophages are induced by co-stimulation of TLR and adenosine A_2__*A*_ receptor agonists and are characterized by high levels of IL-10 and vascular endothelial growth factor and low levels of TNF and IL-12. M2d phenotype macrophages differ from those of M2a, 2b and 2c subtypes due to the absent expression of mannose receptor (CD206) (Colin et al., 2014). Other macrophage phenotypes have been gradually reported in recent years (Figure 1). In contrast to the above macrophages that induced by Th1 and Th2 cytokines, the M4 macrophages induced by platelet chemokine CXCL-4 are irreversible (Domschke and Gleissner, 2019). M4 macrophages are associated with atherogenic and plaque instability due to the production of pro-inflammatory cytokines, such as IL-6 and TNF-α, and the decreased expression of the atheroprotective enzyme heme oxygenase-1 as well as reduced phagocytic properties (Gleissner et al., 2010; Domschke and Gleissner, 2019). Mox macrophages are induced by oxidized low-density lipoprotein (LDL) (ox-LDL) via nuclear factor E2-related factor 2 (Nrf2) in mouse (Kadl et al., 2010). Plaque neovascularization, increased microvessel permeability and leakage of blood can cause intraplaque hemorrhage which induces erythrocyte lysis and release of free hemoglobin. Ingestion of hemoglobin/haptoglobin complexes by CD163 induces differentiation of macrophages into an M (Hb) subtype (Landis et al., 2013). Due to the increased expression of liver X receptor (LXR) α which induces cholesterol efflux, M (Hb) macrophages are characterized by low levels of lipid accumulation (Finn et al., 2012). Furthermore, haem induces monocytes polarization toward Mhem phenotype via activating 5′-AMP-activated protein kinase (AMPK) and the downstream transcription factor 1 and the following heme oxygenase-1 and LXRβ pathway (Chinetti-Gbaguidi et al., 2011; Boyle et al., 2012). Furthermore, enhancers play crucial roles in macrophage development and function as recently reviewed by Hoeksema and Glass (2019).

Single-cell technologies allow precise measurement of individual cell phenotypical and functional variations (Tanay and Regev, 2017). These advanced technologies, such as single-cell RNA sequencing, have further improved our knowledge of macrophage phenotypic diversity (Cochain et al., 2018; Winkels et al., 2018; Willemsen and de Winther, 2020). Furthermore, these advanced technologies raise the possibility for identification and/or monitoring macrophage subtypes in the dynamic atherosclerotic plaques (Willemsen and de Winther, 2020). For instance, triggering receptor expressed on myeloid cells 2 (TREM)^*hi*^ macrophages are recently found in both mouse and human plaque macrophages (Cochain et al., 2018). Combined proteomic and transcriptomic single-cell analysis may change our understanding of macrophage phenotypic diversity and the related regulatory mechanisms in atherosclerotic plaque microenvironment, thereby contributing to a potential therapy for atherosclerosis.

According to a recent study, macrophages (defined asCD68^+^CD11b^+^CD64^+^Ly6C^–^) account for over 50% of the total CD45^+^ cells. The majority of the aortic macrophages are CD206^+^CD169^+^ (markers usually associated with resident macrophages), which could be further separated into 2 subtypes based on the expression of CD209b. CD11c^+^ (with a pro-inflammatory role) and CD206^*lo–int*^ subtypes are the third and fourth most abundant macrophages, respectively. F4/80^*high*^CD11b^*high*^ subtype is the fifth abundant macrophages. Of note, high-fat diet significantly increased the CD11c^+^ macrophages and decreased the CD206^+^CD169^+^CD209b^–^ and CD206^+^CD169^+^CD209b^+^ macrophages in the aortas of apolipoprotein (apo) E-deficient mice, suggesting that high-fat diet may promote inflammatory monocyte-derived macrophages (Cole et al., 2018). In combination with PhenoGraph, CD206^+^CD169^+^CD209b^–^ macrophages can be further divided into 4 clusters (clusters 2, 11, 17 and 20), CD206^*lo–int*^ macrophages are differentiated into cluster 3 and cluster 10, and CD11c^+^ are divided into two clusters based on the expression of MHCII (Cole et al., 2018). Recently, Zernecke et al. (2020) made a meta-analysis of leukocyte in atherosclerotic mouse aortas by comparing the data from 9 single-cell RNA sequencing and 2 mass cytometry studies. Based on these data, 5 subtypes of macrophages are distinguished in atherosclerosis. (1) Resident-like macrophages (expressing genes, such as Lyve1 and Mrc1) are associated with resident aortic macrophages. Of note, these cells also express Pf4 gene, which is used to be considered as platelet specific. (2) Foamy TREM2 macrophages are predominantly found in atherosclerotic aortas and almost absent in healthy aortas. These cells express high levels of MMP12, MMP14, and markers of lipid loading. However, they have low inflammatory gene expression and are suggested to be downstream control of cholesterol metabolism in phagocytes by a TREM2-apoE pathway (Zernecke et al., 2020). (3) Inflammatory macrophages, also designated as chemokine^*high*^ macrophages or non-foamy macrophages, are supposed to be derived from circulation monocytes and display a high levels of proinflammatory genes, such as TNF and IL1b as well as chemokines CXCL1, CXCL2, CCL2, CCL3, and CCL4 (Cochain et al., 2018; Kim et al., 2018; Lin et al., 2019). (4) IFN-inducible macrophages are a small cluster cells that expressing IFN-inducible genes, such as IFIT3, IRF7, and ISG15 (Cochain et al., 2018; Zernecke et al., 2020). These cells may be related to atherosclerosis progression (Chen H. J. et al., 2020). (5) Cavity macrophages only represent a small cell cluster, and they display similarities to monocyte-derived CD226^+^CD11c + MHCII^+^ macrophages (Kim et al., 2016). Presently, the roles of cavity macrophages are not known. The use of single cell RNA sequencing and mass cytometry in conjunction with antibody has improved the identification resolution of macrophage subtypes, but the functional characterization of these subtypes is still far from clear (Honold and Nahrendorf, 2018).

Although M1 and M2 macrophages can be induced *in vitro*, the actual situations *in vivo* are completely different as we discussed above and reviewed by other groups recently (Davies and Taylor, 2015; Zernecke et al., 2020). The complex signals *in vivo* may change the activation process and the final outcomes. *In vivo*, tissue-resident macrophages and monocyte-derived macrophages may exert similar or distinct functions depending on the specific microenvironment as recently reviewed by different groups (Lahmar et al., 2016; Udalova et al., 2016; Italiani and Boraschi, 2017). For instance, during chronic inflammation induced by obesity, monocyte-derived macrophages are recruited to inflamed tissues, where they produce proinflammatory cytokines and exacerbate inflammation. However, the Ron receptor tyrosine kinase expressed on tissue-resident macrophages can reduce inflammatory macrophage activation and promote a repair phenotype (Yu et al., 2016; Allen et al., 2017). Recently, Honold and Nahrendorf (2018) reviewed the ontogeny, function, and interplay of tissue-resident and monocyte-derived macrophages in various organs contributing to CVD. Collectively, the main functions of monocyte-derived macrophages are associated with inflammation and macrophage replenishment, contributing to the accumulation of M1 macrophages. Tissue-resident macrophages are responsible for surveillance, protect host against infection and maintain tissue microenvironment homeostasis, thereby mainly contributing to an M2-like macrophages (Italiani and Boraschi, 2014, 2017; Lahmar et al., 2016; Udalova et al., 2016; Trzebanski and Jung, 2020). However, the functional differences between tissue-resident macrophages and monocyte-derived macrophages are still far from clear.

## Macrophage Distribution in the Atherosclerotic Plaques

**FIGURE 2:**
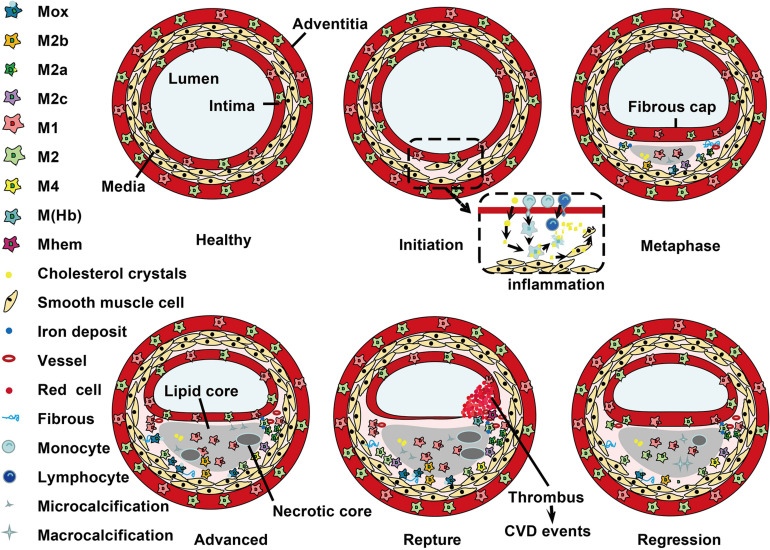
Localization of macrophage subtypes in human atherosclerotic lesions. Atherosclerosis is initiated with a local inflammatory response and the presence of monocyte-derived macrophages. In the intima, macrophages (mainly M1 phenotype) scavenge lipoprotein particles and become foam cells. The secreta of foam cells and M1 macrophages promote lipoprotein retention and sustain inflammation, leading to a further M1 macrophage polarization during atherosclerosis progression. The cholesterol crystals deposited in the plaque can further promote M1 polarization. However, fibrous caps contain similar amounts of M1 and M2 macrophage. In advanced lesions, the gradual accumulation of apoptotic debris results in formation of a necrotic core, which triggers further inflammation and necrosis. Of note, M1 macrophages are predominantly found in the plaque shoulder and lipid core, whereas M2 macrophages localized in the adventitia and areas of neovascularization or outside the lipid core. The degradation of the extracellular matrix and cycles accumulation of lipids may induce plaque rupture and the following thrombosis. In vulnerable plaques, the number of M1 macrophages is increased, while the number of M2 type macrophages is decreased. It is worth noted that M2 macrophages can phagocytose apoptotic M1 macrophages, contributing to the resolution of inflammation. In regressing plaques, there are more M2 macrophages than M1 type macrophages.

Histological analysis has demonstrated that macrophage subtypes distribute at special locations during development of atherosclerosis (Stöger et al., 2012; Peled and Fisher, 2014). The dynamic changes of macrophage phenotype distribution are shown in Figure 2. In humans, M1 and M2 macrophages are present throughout atherogenesis. M1 macrophages dominate the infarction and rupture-prone shoulder regions of the plaque, while the presence of M2 macrophages is associated with vascular adventitia and stable plaques (Stöger et al., 2012; de Gaetano et al., 2016). Furthermore, M2 macrophages in the adventitia are suggested to have migrated from perivascular adipose tissue (Colin et al., 2014). Of note, the comparably stable fibrous caps have equal number of M1 and M2 macrophages (Stöger et al., 2012). In mouse, M2 macrophages are present at the early stages of atherosclerotic plaques, and M1 macrophages are the dominant phenotype in the advanced lesions and reduced in plaque regression (Khallou-Laschet et al., 2010; Peled and Fisher, 2014). It is suggested that the switch between M1 and M2 macrophages is due to conversion of local cells in the plaque (Khallou-Laschet et al., 2010). During the progression of plaques, M1 macrophages are dominant in vulnerable plaques, whereas activation of STAT3/6 promotes the polarization of macrophages to M2 phenotype and lead to atherosclerosis regression (Gong et al., 2017).

Within the advanced plaques, M1 macrophages are mainly localized near the lipid core, while M2 macrophages are mainly clustered in neo-angiogenic areas. These distinct distributions can be partially explained by their distinct biological functions that mainly characterized by the special receptors and signaling molecules expressed on these macrophages. Compared to M1 macrophages, M2 macrophages display low cholesterol handling capacity in line with a reduced expression of LXRα and its target genes ATP-binding cassette (ABC) transporter A1 and apoE. However, M2 macrophages display a high phagocytic activity which is in consistent with the activation of peroxisome proliferators-activated receptors (PPAR) γ (Chinetti-Gbaguidi et al., 2011). For instance, M2a macrophages localize in areas of neovascularization and stable lesion areas. These cells have a high phagocytotic activity (Chinetti-Gbaguidi et al., 2011). M4 macrophages mainly express in the adventitia and intima, and they are associated with atherogenic and plaque instability (Erbel et al., 2015; Domschke and Gleissner, 2019). In hypercholesterolemic mice, Mox macrophages account for ∼30% of plaque macrophages, while M1 and M2 phenotype account for ∼40 and 20% of the remaining macrophages, respectively (Kadl et al., 2010). The cardio-protective M (Hb) and Mhem macrophages may coexist in areas of neovascularization or hemorrhage in atherosclerotic plaques and share some similar atherosclerosis protective properties, such as low ROS production and high cholesterol efflux capacity (Boyle et al., 2011; Chinetti-Gbaguidi et al., 2011). It is possible that M (Hb) and Mhem macrophages are produced due to a complementary mechanism upon stimuli of the atherosclerotic plaque microenvironment. Collectively, M2 phenotype macrophages are not always protective, and the balance between M1 and M2 macrophages is important for the stability of atherosclerotic plaques (Guo L. et al., 2018; Pourcet and Staels, 2018).

## Role of Macrophage in a Dynamic Atherosclerotic Plaque Microenvironment

Previously, plaque macrophages are thought to be derived from monocytes due to the number of circulating monocytes is correlated with the number of macrophages in the atherosclerotic plaque (Swirski et al., 2006). However, accumulating evidence have demonstrated that resident macrophages can proliferate under microenvironment stimuli and dominate plaque macrophage accumulation in all stages of atherosclerosis (Robbins et al., 2013; Lhoták et al., 2016). The atherosclerotic plaque microenvironment, such as inflammation, hyperlipidemia, oxidized lipids, cytokines, endoplasmic reticulum (ER) stress, and other factors, can influence the activation, polarization and function of macrophages, which will in turn influence the plaque microenvironment.

### Macrophage and Inflammation

Atherosclerosis is a chronic inflammatory disease (Ross, 1999; Libby, 2002). Present data suggest that the ratio of pro-inflammatory M1 and anti-inflammatory M2 macrophages, rather than the number of macrophages, within the atherosclerosis plaques may determine the progression and regression of atherosclerosis. The earliest type of lesion, fatty streak, is a pure inflammatory lesion consisted of monocyte-derived macrophages and T lymphocytes (Stary et al., 1994). Afterward, endothelium is activated to secrete chemokines and express adhesion molecules, such as monocyte chemotactic protein 1 (MCP-1), ICAM-1, and VCAM-1, which further attract and bind monocytes in areas of arteries that are prone to permeation (Lusis, 2000). Monocyte-derived macrophages ingest the retained apoB-containing lipoproteins, promoting the intracellular accumulation of lipids (e.g., cholesterol, oxysterols, and fatty acids) as well as inflammatory response (Lusis, 2000; Glass and Witztum, 2001). Of note, the intimal non-foam macrophages mainly contribute to the expression of inflammatory transcripts in atherosclerotic plaques (Kim et al., 2018). Due to the increased expression of migration-inhibitory molecules, such as netrin-1, macrophage emigration from atherosclerotic plaques is reduced, maintaining the inflammatory state of the plaque and contributing to atherosclerosis progression and the formation of complicated and rupture-prone plaques (van Gils et al., 2012; Libby et al., 2014; Barrett, 2020).

Within the plaques, there is a broad range of chemokines, growth factors, differentiation factors, and cytokines, produced by the local lesion and those from the circulation, regulating the phenotype and polarization of macrophages (Wolfs et al., 2011; Ramji and Davies, 2015). Granulocyte-macrophage colony stimulating factor (GM-CSF) and M-CSF mediate the polarization of M1 and M2 macrophages, respectively (Wolfs et al., 2011). The pro-inflammatory cytokines such as TNF and IFN-γ promote M1 polarization and further enhance accumulation of the pro-inflammatory cytokines in the plaques (Ramji and Davies, 2015). On the contrary, anti-inflammatory cytokines, such as IL-4 and IL-10, mediate polarization toward M2 macrophages that generally show an anti-atherosclerosis effect (Nagenborg et al., 2017). Th2 cell-secreted molecules, such as IL-4 and PPARγ, can induce the differentiation of macrophages toward M2 phenotype (Chawla et al., 2001; Laurat et al., 2001). Heme oxygenase-1 is important for the anti-inflammatory activities of M-CSF-polarized M2 macrophages (Sierra-Filardi et al., 2010), whereas Krüppel-like factor (KLF) 2 is involved in the switch of M2 macrophages from anti-inflammatory to pro-inflammatory state (van Tits et al., 2011). The shift of macrophages from M1 to M2 phenotype makes it possible to maintain a basal anti-inflammatory environment in atherosclerosis plaques. However, haptoglobin activated CD163^+^ macrophages that mainly present in atherosclerotic plaques worsen atherosclerosis, despite their anti-inflammatory effects (Guo L. et al., 2018; Pourcet and Staels, 2018).

Reactive oxygen and nitrogen species generated by M1 macrophages may worsen oxidative stress in the plaque, contributing to the deterioration of atherosclerosis (Adamson and Leitinger, 2011). Minimally oxidized LDL induces generation of ROS through activation of TLR4 and thereby stimulates expression of proinflammatory cytokines such as IL-1β and IL-6 (Bae et al., 2009). It is suggested that junction adhesion molecule-like protein is required for ox-LDL-induced up-regulation of macrophage inflammation (Sun et al., 2019). Ox-LDL-CD36 complex triggers TLR2 signaling and promote the pro-inflammatory microenvironment and induce apoptosis of macrophages (Xu et al., 2019). Furthermore, oxidized phospholipids reprogram cellular metabolism and boost hyperinflammation in macrophages (Di Gioia et al., 2020). For instance, they activate transcription factor Nrf2, thereby promoting the differentiation of Mox macrophages, which express redox-regulatory genes as well as pro-inflammatory genes via TLR2 and display a proatherogenic ability (Kadl et al., 2010; Adamson and Leitinger, 2011). Interaction of oxidized lipids with pattern recognition receptors drive macrophages polarization from M2 toward an inflammatory M1 phenotype (Adamson and Leitinger, 2011). Additionally, inflammatory factors can upregulate GM-CSF thereby contribute to the activation of M1 macrophages (Di Gregoli and Johnson, 2012; Newby, 2016). In M2 macrophages, ox-LDL suppresses the expression of KLF2, a nuclear transcription factor known to suppress inflammation, thereby enhancing the production of pro-inflammatory cytokines such as IL-6 and MCP-1 (van Tits et al., 2011).

### Macrophage and Lipids

Macrophages have a fine-tuned system to keep cholesterol balance within the cells and the underlying mechanisms have been reviewed by Yu X. H. et al. (2013). Cholesterol homeostasis in macrophages is important for the progression of atherosclerosis. Lipid-loading modulates cell surface signaling molecules as well as gene expression in cells (Getz and Reardon, 2015). Macrophages in the atherosclerotic plaques ingest apoB-containing lipoproteins (enriched of cholesterol) and turn to foam cell (Chinetti-Gbaguidi et al., 2011; Moore and Tabas, 2011). Hypercholesterolemia can promote myelopoiesis of the monocytes and proliferation of tissue-resident macrophages in the vasculature, thereby accelerating macrophage accumulation in the intimal space (Moore and Tabas, 2011; Hoeksema and Glass, 2019). Of note, cholesterol crystals in the atherosclerotic plaques can induce differentiation of M1 macrophages (Duewell et al., 2010). Macrophage numbers increase up to 20-fold within mouse aorta during atherogenesis (Ylä-Herttuala et al., 1989). Surprisingly, anti-inflammatory M2 macrophages are more susceptible to foam cell formation than M1 macrophages (van Tits et al., 2011). These foam macrophages express many lipid-processing genes and low levels of inflammatory genes compared to the non-foam macrophages (Kim et al., 2018). A mathematical model used for describing lipid accumulation in macrophages and the subsequent changes was established recently (Ford et al., 2019).

Within the atherosclerotic plaques, cholesteryl esters derived from lipoproteins are hydrolyzed in lysosomes via the lysosomal acid lipase, and the produced cholesterol can be re-esterified by acyl-CoA: cholesterol acyltransferase-1 in the ER and then be stored as lipid droplets. Of note, cholesterol at the plasma membrane of macrophages can be removed via ABCA1 and ABCG1 transporters (Chinetti-Gbaguidi et al., 2011; Gao et al., 2019; Xia et al., 2021). M (Hb) and Mhem macrophages express high levels of LXR-α mediating cholesterol efflux and low levels of scavenger receptors involved in lipid uptake, contributing to the low levels of lipid accumulation when compared to that of foam cells (Boyle et al., 2012; Finn et al., 2012). In human atherosclerotic plaques, M2 macrophages display a reduced capacity to handle intracellular cholesterol efflux than M1 macrophages (Chinetti-Gbaguidi et al., 2011). For instance, M2a macrophages localize in areas of neovascularization as well as stable lesion areas away from the lipid core. These cells display low levels of lipid accumulation and cholesterol efflux capacity due to the reduced expression of LXRα and ABC transporters (Chinetti-Gbaguidi et al., 2011). An excessive accumulation of cholesterol in macrophage lysosomes may impair cholesterol efflux capacity and promote the formation of cholesterol crystals in the atherosclerotic plaques.

Different forms of cholesteryl esters may induce macrophage polarization toward M1 phenotype via distinct mechanisms. Cholesteryl linoleate, the major cholesteryl ester in atherosclerotic plaques, induces M1 polarization via a TLR4/NF-κB dependent mechanism. Oxidized cholesteryl linoleates activate macrophages via TLR4, they also stimulate endothelial cells to bind monocytes via extracellular signal-regulated kinase (ERK) 1/2 pathway and induce NF-κB activation in macrophages (Huber et al., 2002; Huang et al., 2010). Of note, many lipids within the plaque microenvironment can promote macrophage polarization toward M2 phenotype. The major oxidized product of cholesteryl linoleate, cholesteryl 9-oxononanoate, induces M2 polarization via a TGF-β signaling pathway, exhibiting a potential anti-inflammatory role (Gargiulo et al., 2009). Conjugated linoleic acid and docosahexaenoic acid induce macrophage polarization toward an anti-inflammatory M2 phenotype, thereby contributing to the regression of atherosclerosis (Titos et al., 2011; McCarthy et al., 2013). The bioactive molecule sphingosine-1-phosphate promotes the production of an anti-inflammatory M2 phenotype macrophages via activating sphingosine-1-phosphate type 1 receptor. However, Sphingosine-1-phosphate displays a proatherogenic effect when interaction with sphingosine-1-phosphate type 2/3 receptors (Hughes et al., 2008). Palmitoylethanolamide reduces M1 phenotype macrophages and promotes the efferocytotic ability of M2 macrophage in mice (Rinne et al., 2018). Unsaturated fatty acids also play an important role in atherosclerosis as recently review by Ménégaut et al. (2019).

### Macrophage Efferocytosis and Apoptosis

In the progression of atherosclerosis, cellular apoptosis and its detrimental effects are partially counterbalanced by phagocytes. Macrophage efferocytosis, termed as their ability to clear apoptotic cells and debris, is an important process for plaque stabilization and resolution of inflammation by reducing necrotic core size (Tabas, 2010a). Macrophages are the dominant phagocytes in atherosclerotic plaques and play a key role in maintaining efferocytosis (Tabas, 2005). In the early stages of apoptosis, cells secrete factors (e.g., lysophosphatidylcholine), termed as “find-me” signals, that attract phagocytes and suppress the secretion of molecules (e.g., CD47), termed as “don’t-eat-me” signals, that normally prevent the phagocytosis of non-apoptotic cells (Tabas, 2010a). Macrophages are responsible for rapidly and efficiently clear cells that have undergone apoptosis in the early atherosclerotic plaques.

In human atherosclerotic plaques, M2 macrophages show higher phagocytosis than M1 macrophages due to the highly expressed opsonins and receptors involved in phagocytosis such as PPARγ (Chinetti-Gbaguidi et al., 2011). MiR-33 skews macrophages toward an M2 phenotype also enhance the efferocytotic capacity and promote plaque regression (Rayner et al., 2011). Mechanistically, macrophage apoptotic cell receptor Mer tyrosine kinase plays a key role in efferocytosis (Cai et al., 2017). For instance, M2c macrophages are more efficient to clear apoptotic cells than other macrophages due to the high expression of Mer tyrosine kinase (Zizzo et al., 2012). Furthermore, reduced lipid metabolism and upregulation of CD47, a key anti-phagocytic molecule, contributing to the decreased efferocytosis in atherosclerotic plaques (Kojima et al., 2016). This defective phagocytic clearance leads to an enhanced inflammation and macrophage apoptosis in advanced atherosclerotic plaques, promoting the formation of necrotic core (Tabas, 2005; Tabas, 2010a; Jinnouchi et al., 2020).

Macrophage apoptosis in early stage benefits atherosclerosis by decreasing the number of the resident cells. Furthermore, the reduced macrophage number may decrease post-apoptotic necrosis and further lesion progression. Of note, apoptotic macrophages are more frequently observed within advanced plaques, especially in the areas that close to the necrotic core (Akishima et al., 2005). ER stress may contribute to macrophage apoptosis in advanced atherosclerotic plaque by activating the expression of the pro-apoptotic protein CEBP-homologous protein (CHOP) (Zinszner et al., 1998; Tabas, 2010b). Knockdown of CHOP reduces ER stress-dependent cell death *in vitro*, and deletion of CHOP protects advanced lesions from apoptosis and plaque necrosis in mice (Thorp et al., 2009; Tsukano et al., 2010). In unstable plaques, macrophage apoptosis triggered by ER stress is regarded as a key step in the formation of necrotic core (Tabas, 2010b). A prolonged ER stress may also activate inflammatory pathways in macrophages, thereby contributing to the progression of atherosclerosis (Tabas, 2010b). Furthermore, macrophage programmed necrotic cell death, such as necroptosis, pyroptosis, and parthanatos, also play key roles in the progression of atherosclerotic plaques (Robinson et al., 2019).

### Macrophage and MMP

Plaques are stabilized by the extracellular matrix produced by SMCs and destabilized by MMP produced by macrophages (Ramji and Davies, 2015). Stable plaques with intact fibrous caps rarely induce detrimental symptoms (Lessner et al., 2004; Chistiakov et al., 2013). However, macrophages facilitate expansive arterial remodeling by increasing the production of MMPs, especially MMP-2 and MMP-9, thereby improving extracellular matrix degradation (Ivan et al., 2002). Plaques with a thin fibrous cap may breakdown from the endothelia and induce thromboembolic events (Virmani et al., 2002; Libby and Theroux, 2005). Therefore, remodeling of the extracellular matrix and cell surface by macrophage-derived MMPs is important for the final outcome of atherosclerotic CVDs.

M1 macrophages upregulate the expression of MMP-1, MMP-3, MMP-10, MMP-12, MMP-14, and MMP-25 depending on mitogen-activated protein kinase (MAPK) and phosphatidylinositol 3-kinase (PI3K). Different chemokines such as TNF-α, GM-CSF, and IL-1β can modulate MMPs via prostaglandin-dependent and -independent mechanisms (Zhang et al., 1998). However, M2 macrophages decrease the expression of MMP-2, MMP-8, and MMP-19, and increase the expression of MMP-11, MMP-12, MMP-25 and tissue inhibitor of MMP type 3. IL-4 selectively induces MMP-12 as well as MMP-25 and tissue inhibitor of MMP type 3 in human monocyte-derived macrophages (Huang et al., 2012). IL-10, an abundant chemokine in atherosclerotic plaques, decreases the expression of MMP-1 and MMP-9 and increases tissue inhibitor of MMP type 1. The different effects of activated M1 and M2 macrophages on the expression of MMPs provide a potential method for modulation of atherosclerotic stability (Huang et al., 2012; Newby, 2016).

Clinically used drugs can modulate macrophage MMPs. The first-line lipid-lowering drugs display attractive activities on inhibition of macrophage MMPs via transcriptional and post-transcriptional mechanisms (Newby, 2008). PPARα agonist can suppress IL-1β-induced MMP-12 production besides lipid-lowering (Souissi et al., 2008). Both PPARα and PPARγ agonists can inhibit MMP-9 secretion from human macrophages (Newby, 2016). Of note, MMP-9 has a dual role in the progression of atherosclerosis. It is suggested that suppressing the production of collagenases, such as MMP-1, MMP-8, MMP-12, and maintaining the activity of MMP-9 which is involved in vascular repair may be a future direction for treating atherosclerosis (Newby, 2005, 2016).

### Macrophage and Vascular Calcification

**FIGURE 3:**
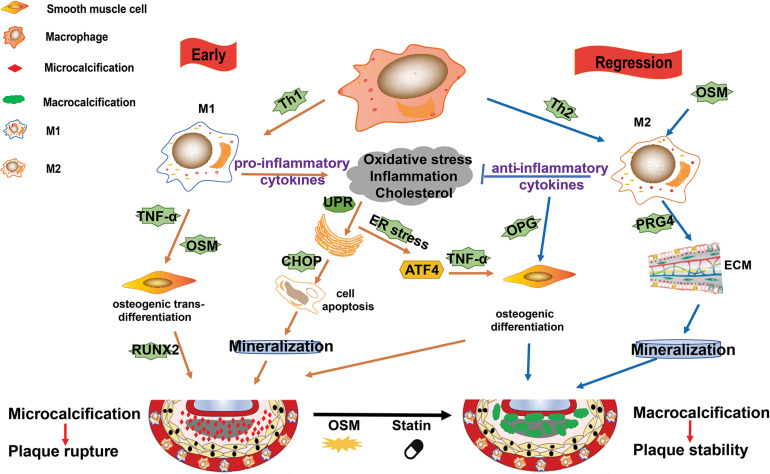
M1/M2 macrophages and their roles in plaque calcification. Calcification occurs in both the intimal and medial layers of the artery. In the plaque microenvironment, multiple factors, such as inflammation, endoplasmic reticulum (ER) stress, osteoblastic differentiation, hyperlipidemia, and oxidative stress, drive the progression of calcification. M1 macrophages mainly contribute to microcalcification by the following ways: (1) M1 macrophages and other cells death (such as induced by ER stress) and the subsequent mineralization; (2) the pro-inflammatory cytokines and Oncostain M (OSM) secreted by M1 macrophages induce osteogenic *trans*-differentiation of vascular smooth muscle cells (VSMCs); and (3) ER stress promotes osteoblastic differentiation of VSMCs. M2 macrophages can induce macrocalcification by the following ways: (1) they counteract inflammation by producing anti-inflammatory cytokines such as IL-10; (2) they promote the production of extracellular matrix; and (3) they enhance osteoblastic differentiation of VSMCs.

Vascular calcification is a hallmark of atherosclerosis. As reviewed by Shioi and Ikari (2018), calcification was ever described as ossification due to its similar mechanism to bone formation. It is acknowledged that calcification occurs in both the intimal and medial layers of the artery. In the plaque microenvironment, multiple factors, such as inflammation, ER stress, osteoblastic differentiation, hyperlipidemia, and oxidative stress, drive the progression of calcification (Shioi and Ikari, 2018; Wang et al., 2018). Macrophages secreted cytokines, such as IL-1β and TNF-α, have been demonstrated to promote atherosclerotic calcification (Shioi et al., 2002; Ceneri et al., 2017). The potential mechanisms of macrophage on promotion of plaque calcification is summarized in Figure 3.

In the early phase, plaque microenvironment induces ER stress that promotes death of the predominant M1 macrophages and the subsequent vesicle-mediated mineralization, contributing to the initial microcalcification (Nadra et al., 2005; Tabas, 2010a). Microcalcification in turn activate inflammation via protein kinase C and MAPK pathways (Nadra et al., 2005; Tabas, 2010a). Furthermore, ER stress can stimulate activating transcription factor (ATF) 4 pathway and promote osteoblastic differentiation of vascular SMCs (VSMCs) (Masuda et al., 2013). Of note, the pro-inflammatory cytokines, such as TNF-α and Oncostain M (OSM), secreted by M1 macrophages may induce osteogenic *trans*-differentiation of VSMCs and promote further mineralization in the plaque lesions (Nadra et al., 2005; Otsuka et al., 2014). This microcalcification cannot form any organized architectures to stabilize the plaque and is associated with increased risk of plaque rupture (Burgmaier et al., 2018; Reith et al., 2018).

It is suggested that the shift of macrophages from M1 to M2 phenotype is important for the resolution of plaque microenvironment (e.g., high levels of inflammation, oxidative stress, and cholesterol) and the regression or stabilization of atherosclerotic plaques. M2 macrophages not only promote anti-inflammatory cytokines and extracellular matrix production and VSMCs maturation, but also accelerate plaque macrocalcification via enhancing osteoblastic differentiation of VSMCs (Shioi and Ikari, 2018). Furthermore, OSM secreted by macrophages may induce M2 polarization and calcification of VSMCs via Janus Kinase (JAK)3-STAT3 pathway, contributing to the atherosclerotic calcification (Shioi et al., 2002; Kakutani et al., 2015). This kind of macrocalcification is believed to form organized structures, thereby stabilizing atherosclerotic plaques (Shioi and Ikari, 2018).

## Potential Therapeutic Strategies Targeting Macrophages in Atherosclerotic Plaque

Atherosclerosis evolves during the lifespan of an individual, and developing therapeutic strategies is an important clinical goal. Clinical trials have demonstrated that cholesterol-lowering statins and proprotein convertase subtillisin/kexin type 9 (PCSK9) inhibitors prevent atherosclerosis progression and promotes plaque regression (Nicholls et al., 2016; Guo et al., 2020). However, lipid-lowering is not sufficient to completely reduce the morbidity and mortality of atherosclerotic CVD and many patients experience adverse cardiac events even with a reached cholesterol-lowering goal (Sabatine et al., 2017; Du et al., 2019; Guo et al., 2020). Macrophages participate in the entire progression of atherosclerosis and have a high plasticity and able to change their phenotypes in response to the microenvironmental stimuli. Therefore, there are a great deal of potential therapeutic strategies via targeting macrophage in the atherosclerotic plaque.

### Targeting Macrophage Polarization

M1 macrophages are dominant in progression lesions while M2 macrophages are enriched in regressing plaques. It is worth noted that a continued recruitment of Ly6C^*hi*^ inflammatory monocytes and their STAT3/6-dependent polarization to the M2 phenotype are required for plaque stabilization and regression in mice (Gong et al., 2017; Rahman K. et al., 2017). These observations suggest that macrophage polarization from M1 phenotype to M2 phenotype may promote atherosclerosis stabilization and regression. More importantly, M1 and M2 macrophages are reversible, which highly raised the possibility of treating atherosclerosis via targeting macrophage plasticity (Sanson et al., 2013).

Different atherosclerotic plaque microenvironment can induce distinct macrophage phenotypes. *In vivo*, the anti-inflammatory humoral factors such as high-density lipoprotein (HDL), apoE, adipopectin and angiotensin converting enzyme, drive M2 polarization (Lovren et al., 2010; Baitsch et al., 2011), while inflammatory factors such as activin A and C-reactive protein (CRP) suppress M2 transformation (Devaraj and Jialal, 2011; Sierra-Filardi et al., 2011). Distinct factors may participate this switch by different mechanisms. Functional HDL particles can suppress inflammation by activating ATF3 and STAT6, which promotes macrophage migration and reverses M1-polarized macrophages to an M2 phenotype (Sanson et al., 2013; Sha et al., 2017). Kallistatin, a plasma protein with anti-inflammatory effect, can inhibit macrophage polarization toward M1 phenotype and promote M2 macrophage polarization via KLF4 activation (Li et al., 2019). IL-19 or recombinant IL-19 induces a reduction in macrophage number and atherosclerotic plaque and an enrichment in M2 macrophages via activating STATs, KLF4, and PPARγ in mice (Gabunia et al., 2016). Bone morphogenetic protein 7 polarizes THP-1 cells into M2 macrophages (Rocher et al., 2012). It is interesting that artificial extracellular matrices composed of collagen I and high sulfated hyaluronan modulate monocytes to M2-like macrophages under sterile inflammation (Kajahn et al., 2012). Furthermore, some iron channels may also contribute to the macrophage polarization. For instance, blocking the calcium-activated potassium channel KCa3.1 inhibits macrophage polarization toward an M1 phenotype and suppresses plaque instability (Xu et al., 2017).

Polarization of macrophages are activated by different chemokines, which activate the transcription factors and signaling pathways through their corresponding receptors. For example, IFN-γ receptor interaction leads to the activation of kinases of the Jak family and then phosphorylation of STAT-1, which induces the expression of genes related to M1 phenotype (Darnell et al., 1994). Deletion of STAT-1 leads to a reduction of the atherosclerotic plaque (Agrawal et al., 2007). Th2 cytokines such as IL-4 and IL-13 can induce STAT6 activation, whereas IL-10 activates STAT3, which then induces macrophage polarization toward M2 phenotype (Lang et al., 2002; Pauleau et al., 2004). Furthermore, IL-4 may induce the polarization of M2 macrophage via inhibiting phosphorylation of ERK and c-Jun N-terminal kinase (Zhao et al., 2016). The phosphatidylinositol 3-kinase (PI3K)-protein kinase B (AKT/PKB) pathway mediates multiple signals and plays important roles in macrophage polarization. It is worth noted that the isoforms of PI3K and AKT have specific functions as recently reviewed by Vergadi et al. (2017). Activation of PPARγ, but not PPARα and PPARβ, is associated with M2 macrophage polarization, while deletion of PPARγ impair this switch (Amine Bouhlel et al., 2007; Bouhlel et al., 2009). MicroRNAs, such as miR155, miR-21 and miR-27b, are expressed in atherosclerotic plaques, where they negatively regulate gene expression by blocking the translation process or by increasing mRNA degradation, thereby modulating macrophage polarization (Stefani and Slack, 2008; Colin et al., 2014; Zhang et al., 2014; Li et al., 2018). Therefore, transcription factors and post-transcriptional regulators play important roles in macrophage polarization. It is possible to control M1 and M2 switch by modulating the related signaling pathways as reviewed by Colin et al. (2014).

Recently, immune-modulatory therapies have been proposed for preventing atherosclerotic CVD (Yamashita et al., 2015). Regulatory T cells (Tregs) can secrete cytokines and stimulate macrophage differentiation via different mechanisms besides Th1 and Th2 (Spitz et al., 2016). For instance, the anti-inflammatory cytokines (e.g., IL-10) secreted by Tregs promote the polarization of M1 macrophages toward M2 macrophages (Kita et al., 2014). Pentoxifylline, an inhibitor of Th1 differentiation pathway, induces the production of IL-10, blocks Th1 polarization and promoting Th2 polarization, thereby contributing a ∼60% atherosclerotic plaque reduction in apoE-knockout mice (Laurat et al., 2001). Furthermore, Tregs play a key role in the regulation of T-cell-mediated immune responses through modulating T-cells proliferation and their secretion of cytokines. Therefore, Tregs are a potential tool for prevention and treatment of atherosclerotic CVDs via regulating macrophage polarization toward M2 phenotype (Foks et al., 2015).

Traditional Chinese medicines play an important role for treatment of atherosclerosis via modulating macrophage activity and polarization. Curcumin, an active ingredient in curcuma rhizomes, inhibits M1 macrophage polarization and the production of pro-inflammatory factors via TLR4/MAPK/NF-κB pathways, and induces M2 macrophage polarization via activating PPARγ, contributing to an anti-atherosclerosis effect (Zhou et al., 2015; Momtazi-Borojeni et al., 2019). Ginsenoside Rb1, one active ingredient of Panax Ginseng, enhances atherosclerotic plaque stability through promoting M2 macrophage polarization (Zhang et al., 2018). Similarly, Ginsenoside Rg3 alleviates atherosclerosis via reversing M1 polarization to M2 polarization in a PPARγ-dependent mechanism (Guo M. et al., 2018). Natural polyphenols also exhibit effect on promoting macrophage switch from pro-inflammatory M1 to anti-inflammatory M2 phenotype (Aharoni et al., 2015). Clinically used drugs and synthesized compounds are a great source for modulating macrophage polarization. For example, metformin can induce primary human monocyte-derived macrophages and mouse bone marrow macrophages toward Mhem phenotype via activating AMPK and its downstream transcription factor 1 and the following heme oxygenase-1 and LXRβ pathway (Boyle et al., 2012; Wan et al., 2013). Sitagliptin inhibits early atherosclerosis via promoting macrophage polarization toward an M2-like phenotype (Brenner et al., 2015). Oleoylethanolamide may suppress M1 macrophage polarization and promote M2 macrophage polarization via AMPK-PPARα pathway (Zhao et al., 2018; Chen Z. et al., 2020). Melatonin, an indoleamine hormone, inhibits M1 polarization and promotes M2 polarization via differentially regulating of AMPKα-STATs pathway in a RORα-dependent manner (Ding et al., 2019). Furthermore, gas signaling molecules such as NO, carbon monoxide (CO), and hydrogen sulfide (H_2_S) may reduce atherosclerosis via modulating macrophage polarization (Yang et al., 2020).

### Targeting Macrophage-Inflammation

The identification of inflammatory biomarkers as independent risk factors for CVD events has promoted the trials using anti-inflammatory strategies for treatment of atherosclerosis (Bäck and Hansson, 2015; Bahrami et al., 2020). Attenuating macrophage-mediated inflammation can promote plaque regression. More importantly, inflammatory cytokine production and release can be modulated by delivering therapeutics into the macrophage cytoplasm or via modulating the related signaling pathways.

In patients with atherosclerosis, MLN1202, a specific monoclonal antibody of CCR2, significantly reduce the level of high-sensitivity CRP (hsCRP), which is a reliable marker of proatherogenic inflammation (Gilbert et al., 2011; Xu et al., 2019). Raising apoA-I and functional levels of HDL improves plaque inflammation and promotes atherosclerosis regression (Barrett et al., 2019; Fotakis et al., 2019). IL-6 contributes to atherosclerotic plaque destabilization, and its antagonist tocilizumab attenuates inflammatory response and the level of hsCRP in patients with non-ST-elevation myocardial infarction (Kleveland et al., 2016). The anti-atherosclerotic and anti-inflammatory effects of AVE0991, a non-peptide angiotensin 1-7 mimetics, maybe attributed to its suppression of pro-inflammatory M1 macrophage differentiation (Skiba et al., 2017). Canakinumab, a monoclonal antibody targeting IL-1β, significantly lower cardiovascular events and hsCRP levels independent of lipid-lowering effect (Ridker et al., 2018). TNF inhibitors have positive cardiovascular effects in rheumatoid arthritis (Roubille et al., 2015). Activation of PPAR-γ pathway or supply IL-13, which enhances anti-inflammatory M2 macrophage polarization, decreases atherosclerosis development in mice (Cardilo-Reis et al., 2012). Additionally, retinoid X receptor-α modulator, K-80003, can attenuate atherosclerotic plaque progression via suppressing autophagy-inflammation axis (Shen et al., 2019). It is proposed that the most attractive approach to modify macrophage polarization is to reduce inflammatory gene expression through RNA interference (Peterson et al., 2018). Loss of AKT2 which favors M1 polarization, promotes anti-inflammatory M2 macrophages and retards atherosclerosis (Babaev et al., 2014). Pro-inflammatory NLRP3 deficient or silence can suppress atherosclerosis and stabilize plaques in mice (Duewell et al., 2010). Regulation of miRNA levels, such as miR21, may control macrophage inflammatory status and the outcome of atherosclerosis (He et al., 2019; Wang H. et al., 2019). Furthermore, immune suppression mediated by Tregs is a potential method for regulating chronic inflammation in atherogenesis. A recent study demonstrated that immunization with macrophage foam cell vaccination results in decreased inflammation and lesion development (Wang F. et al., 2019). Two randomized placebo-controlled clinical trials evaluating anti-inflammatory agents have been conducted in the United States and Canada to clarify whether targeting the inflammation itself will reduce CVD (Yamashita et al., 2015).

Of note, LXRs have anti-inflammatory effects in macrophages and the underlying mechanisms are related to the downregulation of NF-κB pathway (Joseph et al., 2003). LXR induces the expression of arginase II, a characteristic of M2 macrophages and an enzyme that prevent inflammatory nitric oxide production (Marathe et al., 2006). Synthetic LXR ligand inhibits the development of atherosclerosis, even after lesions have been established in mice. However, the synthetic LXR ligands induce hypertriglyceridemia via activating sterol regulatory element-binding protein 1c (Schultz et al., 2000). For this reason, these compounds are not translated well. Desmosterol found in atherosclerotic plaques is an endogenous LXR agonist. The desmosterol mimetics that specifically target LXR pathways in macrophages provide a novel therapeutic strategy (Muse et al., 2018). Furthermore, clinically used drugs play important roles in modulating macrophage inflammation. Pioglitazone induces anti-inflammatory macrophage markers, suggesting a role in stimulating anti-inflammatory M2 macrophage polarization (Feig et al., 2011). Rivaroxaban reduces atherosclerotic plaque progression partially by inhibiting pro-inflammatory activation of macrophages (Hara et al., 2015). Natural products tanshinone IIA and astragaloside IV can reduce macrophage inflammation via suppressing TRL4/NF-κB pathway (Wang et al., 2020). Fucoidan may also have potential effect on macrophage inflammation (Yin et al., 2019b).

### Targeting Macrophage-Mediated Lipid Homeostasis

Macrophage scavenger receptors, such as scavenger receptor A, CD36, LDL receptor-related protein 1 (LRP1) and lectin-like oxidized LDL receptor-1, mediate the uptake of lipids (Robbins et al., 2013). Inhibition of these receptors may reduce lipid accumulation in macrophages. For example, deletion of LRP1 in macrophages are defective in internalizing LDL and accumulation of cholesterol esters *in vivo* (Lillis et al., 2015). To maintain lipid homeostasis, one of the major functions of macrophages in atherosclerotic plaque is the handling of cholesterol and other lipids. A previous study suggested that drugs dissolve or prevent the formation of cholesterol crystals may stabilize fragile plaques (Duewell et al., 2010). Furthermore, ox-LDL activated LXRs promote the outflow of cholesterol via upregulating transporters, such as ABCA1 and ABCG1, in macrophages, thereby alleviating atherosclerosis (Spann et al., 2012). On the contrary, mice deficient in ABCA1 and ABCG1 accelerate atherosclerosis (Yvan-Charvet et al., 2010). Knockdown of miRNAs, such as miR33, that negatively regulating ABC transporters may benefit cholesterol efflux and retard atherosclerosis (Price et al., 2019). HDL and apoA-I are important mediators of reverse cholesterol transport (Paul et al., 2019; Yin et al., 2019a). ApoA-I serves as a major regulator of foam cell lipidome and may play a key role in reducing atherogenic lipid species (Paul et al., 2019). Patients with diabetes mellitus have a two to fourfold higher risk of atherosclerotic CVD due to impaired polarization of plaque macrophages to M2 phenotype as well as lipid lowering (Parathath et al., 2011). Raising functional HDL in diabetic mice promotes plaque levels of M2 macrophage and enhances the cholesterol efflux capacity as well as anti-inflammatory functions (Barrett et al., 2019). Of note, the desmosterol mimetics that specifically targeting LXR pathways can increase macrophage cholesterol efflux and enhance the ability of macrophages to emigrate, providing a potential novel therapeutic strategy (Muse et al., 2018). Our previous studies demonstrated the marine-derived or natural compounds can modulate macrophage lipid homeostasis *in vitro* (Li et al., 2020a, b) and promote cholesterol reverse transport *in vivo* (Yang et al., 2019; Yin et al., 2019a). PPARα and PPARγ agonists may also promote macrophage cholesterol efflux via activating ABCA1 expression (Chinetti et al., 2001; Li et al., 2004).

### Macrophage-Targeted Drug Delivery

**FIGURE 4:**
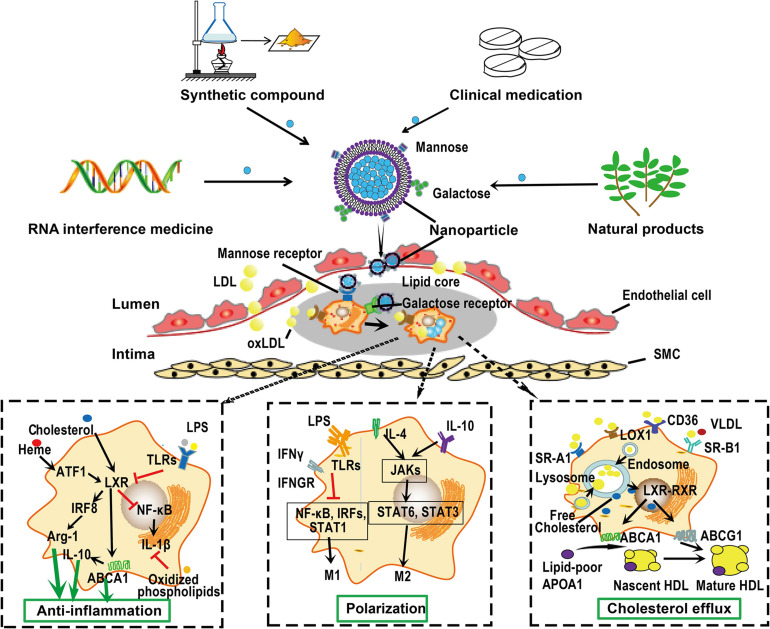
Macrophage-targeted drug delivery. Macrophages can endocytose particles from nanometers to micrometers in size. Furthermore, distinct macrophage phenotypes express distinct cell surface receptors that can bind special ligands. This raise the possibility of delivering drugs targeting atherosclerotic plaque macrophages. Drugs or siRNA can be carried by nanoparticles, whose surface are specifically modified by ligands, such as mannose and galactose, that can be recognized by receptors on the surface of macrophages. These nanoparticles can be captured by macrophages on the surface or within the atherosclerotic plaques. Once in position, these nanoparticles can be disassembled and drugs are released to exert their functions such as anti-inflammation, promoting macrophage polarization and cholesterol efflux. Given the monotherapy of lipid-lowering drugs could not completely retard the progression of atherosclerosis, these nanoparticles may be designed to carry several compounds with different functions, thereby promoting the efficiency of treatment. ABC, ATP-binding cassette; ATF, activating transcription factor; IRF, interferon regulatory factor; JAK, Janus kinase; LDL, low-density lipoprotein; NF-κB, nuclear factor-kappa B; RXR, retinoid X receptor; SMC, smooth muscle cell; SR-A, scavenger receptor A; STAT, signal transducers and activators of transcription; VLDL, very low-density lipoprotein.

Macrophages are particularly important to be considered as therapeutic targets for treatment of atherosclerosis because they participate the whole progression of atherosclerosis and display a great plasticity upon stimuli as discussed earlier. Macrophages are present in all vertebrate tissues with distinct functions and cell surface markers that could ultimately be used for tissue specific macrophage targeting (Gordon and Plüddemann, 2017). Some receptors, such as CD68 (human), F4/80 and CD11b (mice), are generally expressed on all macrophages (Murray and Wynn, 2011; Gordon and Plüddemann, 2017). More importantly, distinct macrophage subtypes may express specific receptors on their membrane surface as we described earlier. Researchers can modify the particle surface to meet the requirement of targeting special macrophage receptors or local atherosclerotic plaques. A proposed scheme for macrophage-targeted drug delivery is shown in Figure 4.

More importantly, macrophages can endocytose particles from nanometers to micrometers in size due to their phagocytic characteristic (Peterson et al., 2018). Recent advances in drug delivery systems, such as micro- and nanoparticles, liposomes, and oligopeptide complexes, provide the potential for selectively target macrophages (Hillaireau and Couvreur, 2009; Peterson et al., 2018). Designed liposomes that carrying monoclonal antibodies of IL-6 and CD163 can be ingested by M1 and M2 phenotype macrophages, respectively (Etzerodt et al., 2012). The high level of mannose receptor expression on M(Hb) and Mhem cells provides a way to target these cells via mannosylated particles. Mannose-functionalized dendrimeric nanoparticles that conjugated LXR agonists can be selectively ingested by macrophages and exert their functions within cells (He et al., 2018). A cylic nonapeptide, LyP-1, displays preferentially affinity for hypoxic atherosclerotic plaques and triggers apoptosis of plaque macrophages in mice (She et al., 2016). A pH-responsive and mannosylated polymeric micelles can successfully achieve CD206 (mannose receptor)-targeted siRNA delivery (Yu S. S. et al., 2013). Beta 1,3-D-glucan-encapsulated siRNA particles are efficient oral delivery vehicles that silence pro-inflammatory or other genes via targeting macrophages (Aouadi et al., 2009). Plaque-hyaluronidase-responsive HDL-mimetic nanoparticles can efficiently target intimal macrophage for drug delivery (Zhang et al., 2017). Hyaluronan nanoparticles selectively target plaque-associated macrophages and reduce inflammation *in vivo* (Beldman et al., 2017). Once in position, various approaches could be applied to modulate macrophages, such as cell apoptosis, anti-inflammatory and cholesterol efflux promoting therapy. For instance, targeted delivery of antioxidant into the atherosclerotic plaques may modify atherosclerosis by attenuating and production of ROS and further oxidation of lipids in the subendothelial space, and the subsequent release of inflammatory cytokines in macrophages (Toledo-Ibelles and Mas-Oliva, 2018; Fiorelli et al., 2019).

## Concluding Remarks and Future Directions

Recent studies have demonstrated that atherosclerosis progression is associated with macrophage phenotypic diversity. As lipid-lowering cannot completely retards the progression of atherosclerosis, macrophages with a great plasticity represent a potential therapeutic target. Therapies that reduce plaque macrophage accumulation, inflammation, and oxidative stress, and especially promote macrophage polarization to an atheroprotective phenotype may benefit the outcomes of atherosclerotic CVDs. Given the important roles of macrophages in atherosclerosis and the advanced drug delivery system, it is reasonable to explore a viable therapeutic strategy targeting atherosclerotic plaque macrophages. However, much work is needed to fully understand this area and to enable a reliable therapeutic strategy targeting the dynamic atherosclerotic plaque microenvironment. (1) Macrophage phenotype and function need to be further clarified using recently developed technologies such as single cell analysis, especially the potential diversity of M1 macrophages. (2) Data from mice cannot directly be translated into humans because macrophage phenotypic diversity as well as the atherosclerotic plaque evolution in mice and humans are inconsistent. Therefore, an increased understanding of the role of macrophages in human atherosclerosis is anticipated. (3) The development of atherosclerosis involves many cells, understanding of the crosstalk between macrophages and other cells in the atherosclerotic plaque need to be improved. (4) Due to the important role of hyperlipidemia in the progression of atherosclerosis, the macrophage-targeted therapeutic strategy should be combined with lipid-lowering therapies.

## Author Contributions

PL, H-HJ, and Y-JL performed reference collection and prepared the manuscript. S-DG re-edited the manuscript. All authors contributed to the article and approved the submitted version.

## Conflict of Interest

The authors declare that the research was conducted in the absence of any commercial or financial relationships that could be construed as a potential conflict of interest.

## Abbreviations

ABC: ATP binding cassette
AKT/PKB: protein kinase B
AMPK: 5′-AMP-activated protein kinase
apo: apolipoprotein
CCR: CC chemokine receptor
CD: cluster of differentiation
CHOP: CEBP-homologous protein
CRP: C-reactive protein
CVD: cardiovascular disease
CXCL: chemokine (C-X-C motif) ligand
ER: endoplasmic reticulum
ERK: extracellular signal-regulated kinase
GM-CSF: granulocyte-macrophage colony stimulating factor
HDL: high-density lipoprotein
hsCRP: high-sensitivity CRP
ICAM-1: intercellular cell adhesion molecule-1
IL: interleukin
JAK: Janus kinase
KLF: Krüppel-liker factor
LDL: low-density lipoprotein
LRP1: LDL receptor-related protein 1
LXR: liver X receptor
MAPK: mitogen-activated protein kinase
MCP-1: monocyte chemotactic protein 1
M-CSF: macrophage colony stimulating factor
MMP: matrix metalloprotease
NF- κ B: nuclear factor kappa-B
NO: nitric oxide
Nrf2: nuclear factor E2-related factor 2
ox-LDL: oxidized LDL
OSM: Oncostain M
PCSK: proprotein convertase subtillisin/kexin type 9
PI3K: phosphatidylinositol 3-kinase
PPAR: peroxisome proliferators-activated receptors
ROS: reactive oxygen species
SMCs: smooth muscle cells
STAT: signal transducers and activators of transcription
TGF-β: transforming growth factor β
Th: T helper
TLR: toll-like receptor
TNF: tumor necrosis factor
IFN: interferon
LPS: lipopolysaccharide
VCAM-1: vascular cell adhesion molecule 1
VSMCs: vascular SMCs.
